# Age-dependent changes in metabolic profile of turkey spermatozoa as assessed by NMR analysis

**DOI:** 10.1371/journal.pone.0194219

**Published:** 2018-03-13

**Authors:** Nicolaia Iaffaldano, Michele Di Iorio, Luisa Mannina, Gianluca Paventi, Maria Pina Rosato, Silvia Cerolini, Anatoly P. Sobolev

**Affiliations:** 1 Department of Agricultural, Environmental and Food Sciences, University of Molise, Campobasso, Italy; 2 Dipartimento di Chimica e Tecnologie del Farmaco, Sapienza Università di Roma, Rome, Italy; 3 Istituto di Metodologie Chimiche, Laboratorio di Risonanza Magnetica “Annalaura Segre”, CNR, Monterotondo, Rome, Italy; 4 Department of Medicine and Health Sciences “V. Tiberio”, University of Molise, Campobasso, Italy; 5 Department of Veterinary Medicine, University of Milan, Milan, Italy; National Research Council of Italy, ITALY

## Abstract

Metabolic profile of fresh turkey spermatozoa at three different reproductive period ages, namely 32, 44 and 56 weeks, was monitored by Nuclear Magnetic Resonance (NMR) spectroscopy and correlated to sperm quality parameters. The age-related decrease in sperm quality as indicated by reduction of sperm concentration, sperm mobility and osmotic tolerance was associated to variation in the level of specific water-soluble and liposoluble metabolites. In particular, the highest levels of isoleucine, phenylalanine, leucine, tyrosine and valine were found at 32 weeks of age, whereas aspartate, lactate, creatine, carnitine, acetylcarnitine levels increased during the ageing. Lipid composition also changed during the ageing: diunsaturated fatty acids level increased from 32 to 56 weeks of age, whereas a reduction of polyunsaturated fatty acids content was observed at 56 weeks. The untargeted approach attempts to give a wider picture of metabolic changes occurring in ageing suggesting that the reduction of sperm quality could be due to a progressive deficiency in mitochondrial energy producing systems, as also prompted by the negative correlation found between sperm mobility and the increase in certain mitochondrial metabolites.

## Introduction

Avian semen is a complex fluid comprising spermatozoa and seminal plasma. Semen quality and, consequently, fertilizing ability depend strongly on semen composition [1, 2] that consists of many compounds, widely ranging in concentration, with different structures and physicochemical properties [3–6]. Chemical composition, as well as sperm quality, can be influenced by various factors such as dietary manipulation, strain and, particularly, ageing [7–10]. Therefore, a deeper knowledge on semen composition and its variation during ageing is needed to improve avian reproduction, farming output and genetic resources diversity preservation [11–13].

In this regard, the metabolite profile of avian spermatozoa and, in particular, its age-related changes have been only partially investigated using limited targeted approaches focalised on specific metabolites or classes of metabolites such as lipids, amino acids, and carbohydrates whose levels affect sperm quality and fertilizing ability, as well as, semen storability [10, 14, 15]. Sperm lipids are involved in vital aspects of avian sperm metabolism and functions: in particular, they contribute in maintaining the plasma membrane integrity, whereas sperm lipid changes following dietary manipulation affect turkey semen quality [10]. Carbohydrates have been reported to be particularly important for structure and function of glycocalyx essential for sperm functions [16]. Moreover, in mammalian sperm, amino acids play an important role in many metabolic processes involved in sperm motility, acrosome reaction and capacitation, whereas, in addition, carnitine is implicated in the energy metabolism of sperm promoting motility, maturation and spermatogenic process [17].

Currently, to the best of our knowledge, a comprehensive metabolite characterization of turkey sperm has not yet been reported. Among the analytical methods applied in metabolite characterization, Nuclear Magnetic Resonance (NMR) is one of the most suitable approach since it allows detection and quantification of a wide range of metabolites simultaneously with a high analytical precision. NMR based approach has already found applications in the analysis of biological fluids of mammals [18, 19], and human [20, 21].

Here, the metabolic profile of fresh turkey spermatozoa at 32, 44 and 56 weeks of the reproductive period was investigated by NMR approach in order to evaluate the age-related changes in the metabolic profile and their influence on the sperm quality.

## Materials and methods

### Materials

The chemicals used in this study were purchased from Sigma Chemical Co. (St. Louis, MO).

### Animals and semen treatment

350 Hybrid Large White turkey males of a private breeding farm (Agricola Santo Stefano of Amadori’s group, Canzano, TE, Italy) were used. Thus, all procedures reported in this work and that contribute to the care and use including the semen collection of Hybrid Large White turkey male (Aviagen turkey) are performed at a commercial Amadori breeding that complies with the ethical standards of the Aviagen guides. Aviagen is a member of European Forum of Farm Animal Breeders (EFFAB) and follows the Code of Good Practice Code-EFABAR and Principles of Sustainable Breeding (http://en.aviagen.com/about-us/welfare-3/). No animal was anaesthetized, mistreated or sacrificed during this study; semen samples were routinely collected as part of the standard management procedure for male turkey breeders at the breeding farm. Birds were housed at 29 weeks and reared up to 58 weeks of age. They were maintained under standard management conditions and photostimulated on a daily basis with a 14L:10D photoperiod. Turkey males were kept in groups of 8–10 in floor pens. Feed and water were provided ad libitum. Animals were trained for semen collection by abdominal massage two times a week.

Semen used in this trial was collected from randomly-selected males when turkeys were at the beginning (32 weeks), in the middle (44 weeks) and at the end of the reproductive period (56 weeks).

Semen was collected by abdominal massage; ejaculates were pooled and thoroughly mixed; each pool originating from a minimum of 9 to a maximum of 12 ejaculates in order to reach at least 4 mL of semen/pool. At each sampling age, 5 pools of semen were realized and used without any selection according to the quantity or quality of semen parameters.

### Semen quality evaluation

The sperm concentration was determined with a Neubauer counting chamber, then semen was four-fold diluted with the Tselutin extender (128 mM Na-glutamate; 20 mM K_2_HPO_4_; 44.4 mM glucose; 11.1 mM inositol; 7 mM Mg-acetate; 13.3 mM glycine; 7.68 mM glutamic acid; pH 6.65). Each diluted semen pool was used for the analysis on fresh semen, as described below.

Sperm mobility was assessed using the Sperm Motility Test (SMT) according to the Accudenz^®^ procedure (Accurate Chemical & Scientific Corp., Westbury, NY 11590) described by Iaffaldano et al. [22] with the Accudenz^®^ concentration suggested by King et al. [23] for turkey semen. The test is based on the ability of the spermatozoa with a forward progressive motility to penetrate a 4% Accudenz^®^ layer. Semen was diluted to 1.0× 10^9^ sperm/mL in 111 mM NaCl buffered with 50 mM N-tris-[hydroxymethyl]methyl-2-amino-ethanesulfonic acid (TES), pH 7.4, containing 25 mM glucose and 4 mM CaCl_2_. A 60 μL sample of each sperm suspension was overlaid onto 600 μL of pre-warmed 4% (w/v) Accudenz^®^ solution in a semi-micro polystyrene disposable cuvette. Cuvettes were incubated for 5 minutes in a 41°C water bath, placed in a spectrophotometer and absorbance was measured at 550 nm after 60 s. The sperm mobility was assessed by values of optical density (O.D.).

Sperm viability was assessed by means of a dual staining technique using the stains SYBR-14 and Propidium Iodide (PI) as described by Iaffaldano et al. [24, 25]. Both fluorescent dies were supplied in the LIVE/DEAD sperm viability kit (Molecular Probe, Eugene, OR, USA). SYBR-14, a membrane-permeant DNA probe stained only the nuclei of living sperm to a fluorescent bright green colour when excited at 488 nm. Nonviable spermatozoa were detected with propidium iodide (PI), which stained the sperm red. The PI was not incorporated into live sperm with intact membranes. The SYBR-14 was first diluted 1:100 into dimethyl sulfoxide (DMSO) while PI was dissolved 1:100 in PBS. Aliquots of 5 μl diluted semen were put in 39 μl of Tselutin diluent containing 1 μl of SYBR. Samples were incubated for 10 min at 37°C, then 5 μl of PI were added in samples that were further incubated at 37°C for 5 min. The evaluation of viable/nonviable spermatozoa was performed using fluorescence microscopy (blue excitation filter λ = 488 nm; ×100 oil immersion objective; magnification ×400). At least 200 spermatozoa were counted. Percentages of viable spermatozoa were calculated as the ratio: green cells/ (green cells + red cells) × 100.

To determine sperm membrane functional integrity known as sperm osmotic tolerance (SOT), an adaptation of the hypo-osmotic H_2_O test described by Donoghue and Donoghue [26] was used: 5 μl aliquots of diluted semen were added to 39 μl of distilled H_2_O and then they were stained with SYBR/PI and read as described above for sperm viability. This test is effective in measure changes in sperm membrane functional status and permeability when exposed to a hypo-osmotic medium such as water. The rationale of this test is based on the assumption that the undamaged sperm membrane permits passage of water into the cytoplasmatic space causing swelling produced by the hypo-osmotic shock but in chemically active sperm the pressure so generated no leads to the rupture of membrane. In this cells only SYBR-14 can enter and they appear coloured green at the fluorescent microscope. Conversely, in damaged sperm the membrane allows water to pass across the membrane without any accumulation and in undamaged but chemically inactive sperm the membrane is unable to survive at the pressure generated by the hypo-osmotic shock and breakages in the membrane occurs that permits passage of PI that stains in red the spermatozoa that have lose their functional integrity.

### NMR measurements

#### Sample preparation

Bligh and Dyer [27] method was used to extract and separate water-soluble and liposoluble metabolites from semen samples. Aliquots of diluted semen containing 15 × 10^9^ spermatozoa were centrifuged at 1500 rpm at 5°C for 15 min to remove diluent and seminal plasma. A chloroform/methanol (2:1, v/v) mixture was added to the sperm pellet and sample was homogenized with a vortex mixer for 60 s before adding distilled water in the proportion of 1:18:4 (spermatozoa:chloroform/methanol:water). The homogenate was centrifuged at a speed of 4000 rpm for 20 min at 5°C. The liquid chloroform and water/methanol phases were separated and dried under vacuum in a rotary evaporator. The dried residues were dissolved in 0.75 mL of CDCl_3_/CD_3_OD (2:3v/v) or 0.75 mL of D_2_O phosphate buffer (400 mM, pD = 7).

#### NMR spectra

The NMR spectra of aqueous and organic extracts were recorded at 27°C on a Bruker AVANCE 600 NMR spectrometer operating at the proton frequency of 600.13 MHz and equipped with a Bruker multinuclear z-gradient inverse probe head capable of producing gradients in the z-direction with a strength of 55 G/cm. ^1^H spectra were referenced to methyl group signals of 3-(trimethylsilyl)-propionic-2,2,3,3-d4 acid sodium salt (TSP, δ = 0.00 ppm, 0.3 mM) in D_2_O, and to the residual CHD_2_ signal of methanol (set to 3.31 ppm) in CDCl_3_/CD_3_OD mixture. ^1^H spectra of aqueous extracts were acquired by coadding 512 transients with a recycle delay of 3 s. The residual HDO signal was suppressed using a standard Bruker presaturation sequence zgpr. The experiment was carried out by using a 45° pulse of 7.25 μs, 32K data points. ^1^H spectra of CDCl_3_/CD_3_OD extracts were obtained using the following parameters: 256 transients, 32K data points, recycle delay of 3 s and a 90° pulse of 10 μs.

The ^1^H spectra were Fourier transformed using exponential multiplication function with line broadening factor of 0.3 Hz, and manual phase correction and baseline correction were applied. This processing procedure was performed using Bruker TOPSPIN software, version 1.3.

The assignment of ^1^H NMR spectra of aqueous and organic extracts of turkey sperm were obtained using 2D experiments, literature data and standards addition, see S1 and S2 Tables in supporting information.

2D NMR experiments, namely ^1^H–^1^H total correlation spectroscopy (TOCSY), ^1^H–_13_C heteronuclear single quantum coherence (HSQC), and ^1^H–^13^C heteronuclear multiple bond correlation (HMBC), were performed using the same experimental conditions previously reported [28]. The mixing time for the ^1^H–^1^H TOCSY was 80 ms. The HSQC experiments were performed using a coupling constant ^1^*J*_C–H_ of 150 Hz and the ^1^H–^13^C HMBC experiments were performed using a delay for the evolution of long-range couplings of 80 ms.

#### Measurement of the metabolite content in aqueous extract

The integral of 22 ^1^H resonances due to water-soluble assigned metabolites (see S1 Table) were measured with respect to the integral of TSP signal (0.00 ppm, 9H, Si(CH_3_)_3_ group) used as internal standard and normalized to 100. Manual integration of selected resonances was performed using Bruker TOPSPIN software version 1.3. Concentrations (in mM) of metabolites in NMR solutions were calculated using the following equation:

$$c_{i}=c_{TSP}\frac{I_{i}}{I_{TSP}}\frac{9}{n_{i}},$$

where c_*i*_ and c_*TSP*_ are concentrations of i^th^ metabolite and TSP, respectively; *I*_*i*_ and I_*TSP*_ are integrals of corresponding signals; *n*_*i*_ is the number of equivalent protons for the selected signal. In the case of glucose, the sum of α- and β- anomers concentrations was calculated. All data are reported in S3 Table in supporting information.

#### Measurement of the metabolic content in organic extracts

The integrals of eight ^1^H resonances due to assigned liposoluble metabolites were measured and used to obtain the molar percentages, see S2 Table. Manual integration of selected resonances was performed using Bruker TOPSPIN software version 1.3. Lipidic fraction consists of free and esterified fatty acids chains, sphingomyelin, and cholesterol. The molar percentages of lipids were calculated taking into account the integrals of selected signals and the corresponding number of equivalent protons according to the following equation:

$$mol\%=\frac{I_{i}/n_{i}}{I_{\alpha }/2+I_{Chol}/3+I_{SMN}}100\%,$$

where *I_i_*, *I_α_*, *I_Chol_*, and *I_SMN_* are integrals of selected signals (*I_α_*, α-CH_2_ groups of all fatty acid chains, *I_Chol_* CH_3_ group of cholesterol, *I_SMN_* CH proton of sphingomyelin); *n_i_* is the number of equivalent protons for the selected signal.

The resonances due to CH_3_ of cholesterol (3H, 0.74 ppm), all allylic protons (UFA) (4H, 2.08 ppm), α-CH_2_ groups of all fatty acid chains (2H, 2.31 ppm), CH_2_ diallylic protons of diunsaturated fatty acids (DUFA) (2H, 2.81 ppm), CH_2_ diallylic protons of polyunsaturated fatty acid (PUFA) (4H, 2.88 ppm), CH_2_N of phosphatidylethanolamine (PE) (2H, 3.21 ppm), (CH_3_)_3_N^+^ of phosphatidylcholine (PC) (9H, 3.28 ppm) CH (double bond) proton of sphingomyelin (SMN) (1H, 5.76 ppm) were integrated. The molar % of all saturated fatty chains (SFA) was calculated as 100-UFA, where UFA were calculated using the all allylic proton signal at 2.08 ppm. All data are reported in S4 Table in supporting information.

### Statistical analysis

Metabolites concentrations calculated by NMR analysis (water and lipid extracts) and sperm quality parameters (concentration, mobility, viability and sperm osmotic tolerance) at different ages were compared by ANOVA, followed by Duncan’s comparison test setting significance, threshold at P < 0.05.

Correlations among sperm variables and metabolites identified by NMR analysis were assessed through Pearson’s correlation coefficients setting significance threshold at the P < 0.05 level (one-tailed) and P < 0.01 levels (two-tailed). All statistical tests were performed using the software package SPSS (SPSS 15.0 for Windows, 2006; SPSS, Chicago, IL, USA).

## Results

### Sperm quality

**Fig 1:**
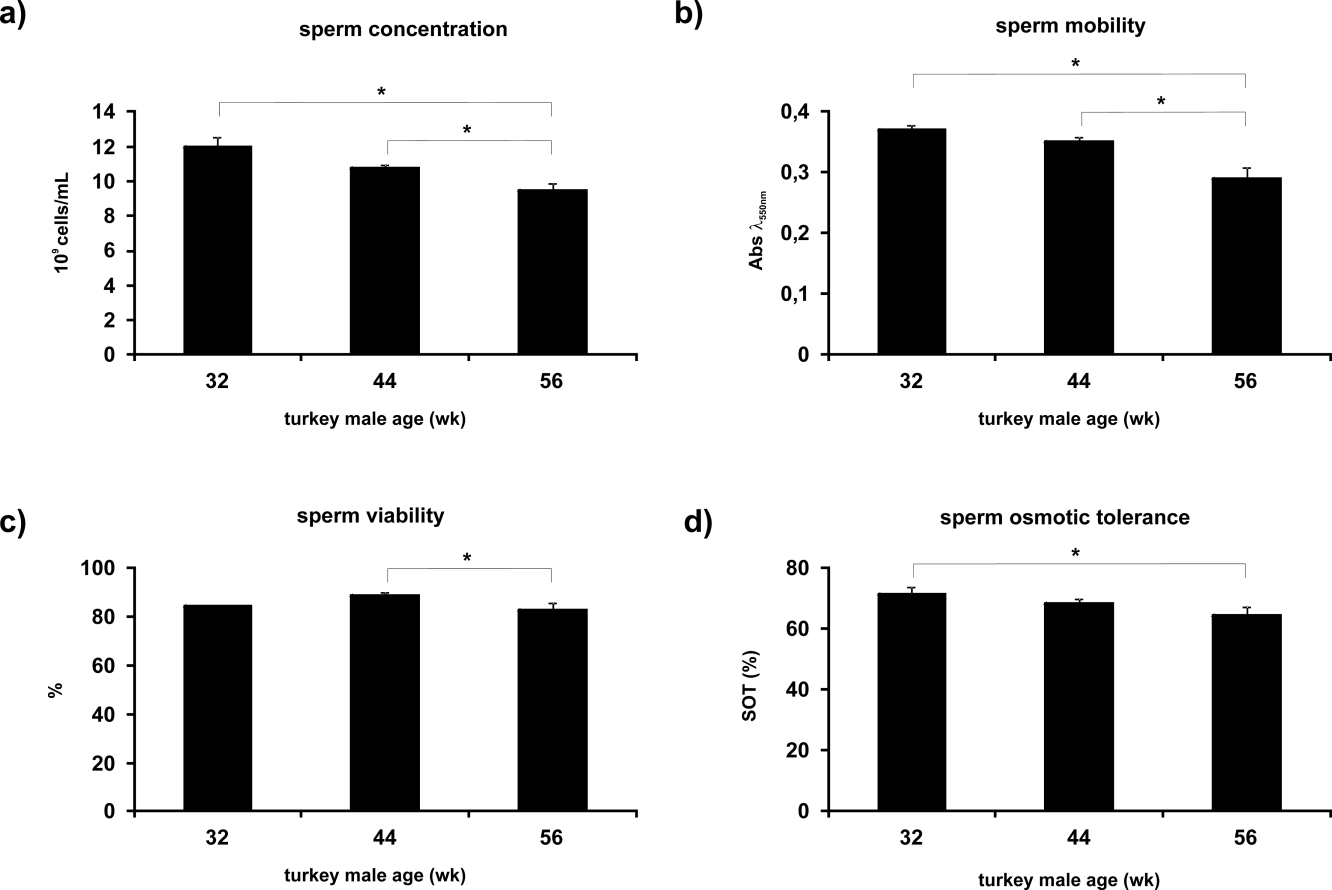
Effect of turkey male ages on sperm quality parameters. Mean values ± SE (n = 5) for sperm concentration (a), sperm mobility (b), sperm viability (c) and SOT (d) were evaluated at 32, 44 and 56 weeks of age. For details see Methods section. * indicates significant differences (P < 0.05).

Sperm concentration, mobility, viability and SOT of semen samples collected from turkey breeders at different weeks of reproductive age are reported in Fig 1. All sperm quality parameters were significantly affected during ageing.

Very high value of sperm concentration was recorded at 32 weeks of age and a progressive decrease was found during ageing; a significant decrease was found at 56 weeks compared to the previous ages (Fig 1A).

A similar trend was observed in sperm mobility: significantly higher values were recorded at 32 and 44 weeks compared to 56 weeks (Fig 1B).

In contrast, sperm viability did not show a clear trend during ageing; a significant decrease in sperm viability was recorded from 44 weeks to 56 weeks, whereas no significant differences were detected between 32 and 44 weeks, and between 32 and 56 weeks (Fig 1C).

SOT showed a progressive significant decrease during ageing. The highest value was measured at the beginning of the reproductive period at 32 weeks of age and a significant decrease was recorded at 56 weeks, whereas an intermediate value was recorded at 44 weeks with no statistical difference compared to the other ages (Fig 1D).

### NMR analysis

**Fig 2:**
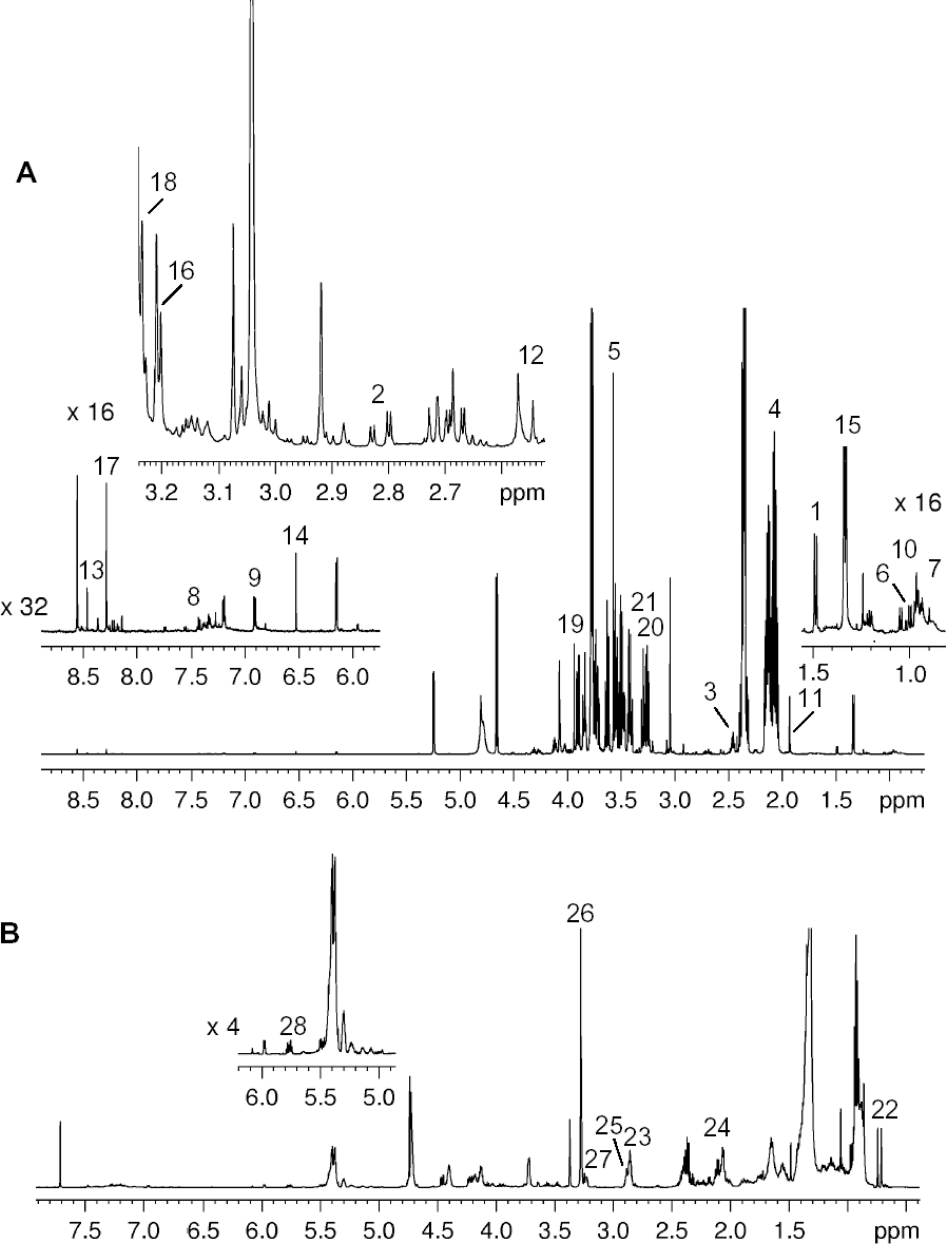
^**1**^**H NMR spectra of aqueous (A) and organic extracts (B) of turkey spermatozoa at 32 weeks of age.** Assignments: 1, Ala; 2, Asp; 3, Gln; 4, Glu; 5, Gly; 6, Ile; 7, Leu; 8, Phe; 9, Tyr; 10, Val; 11, Acetate; 12, Citrate; 13, Formate; 14, Fumarate; 15, Lactate; 16, Ac-carnitine; 17, AMP; 18, Carnitine; 19, Creatine; 20, Glucose; 21, Myo-inositol; 22, CHO; 23, DUFA; 24, UFA; 25, PUFA; 26, PC; 27, PE; 28, SMN. For details see Methods section.

NMR based turkey sperm metabolite profiling allows all identified metabolites to be included in the analysis without any bias towards to particular classes of compounds. The number of metabolites is restricted only by the detection limit of the analytical technique [29]. In Fig 2 ^1^H NMR spectra of aqueous and organic extracts of turkey sperm are shown together with the assignments. The spectra at three different ages (data not reported) show the same signals with different intensities. This means that the same metabolites are present although at different concentrations.

All identified metabolites quantified at three different ages are reported in Table 1.

**Table 1:** Metabolites identified by NMR in fresh spermatozoa from 32, 44 and 56 weeks old turkey male. Mean values ± SE.

Metabolite, ^1^H chemical shift (ppm)	32 weeks	44 weeks	56 weeks
**Water extract**			
***Amino acids***	**c, mM (n = 5)**	**c, mM (n = 4)**	**c, mM (n = 5)**
Ala (1.48)	0.300 ± 0.011 ^a^	0.346 ± 0.006 ^a^	0.298 ± 0.026 ^a^
Asp (2.83)	0.398 ± 0.008 ^a^	0.526 ± 0.022 ^ab^	0.688 ± 0.096 ^b^
Gln (2.45)	2.858 ± 0.726 ^a^	2.742 ± 0.302 ^a^	2.558 ± 0.393 ^a^
Glu (2.07)	113.263 ± 7.648 ^a^	111.515 ± 10.694 ^a^	133.387 ± 16.000 ^a^
Gly (3.57)	7.936 ± 0.621 ^a^	8.146 ± 0.896 ^a^	9.778 ± 1.266 ^a^
Ile (1.02)	0.025 ± 0.002 ^a^	0.016 ± 0.001 ^b^	0.017 ± 0.003 ^b^
Leu (0.96)	0.091 ± 0.004 ^a^	0.055 ± 0.003 ^b^	0.056 ± 0.009 ^b^
Phe (7.43)	0.037 ± 0.001 ^a^	0.028 ± 0.001 ^b^	0.026 ± 0.003 ^b^
Tyr (6.92)	0.080 ± 0.005 ^a^	0.040 ± 0.008 ^b^	0.044 ± 0.008 ^b^
Val (0.99)	0.063 ± 0.004 ^a^	0.048 ± 0.001 ^b^	0.049 ± 0.005 ^b^
***Organic acids***			
Acetate (1.93)	0.858 ± 0.159 ^a^	0.596 ± 0.225 ^a^	0.593 ± 0.220 ^a^
Citrate (2.57)	0.152 ± 0.017 ^a^	0.225 ± 0.013 ^a^	0.221 ± 0.051 ^a^
Formate (8.46)	0.068 ± 0.009 ^a^	0.133 ± 0.045 ^a^	0.119 ± 0.033 ^a^
Fumarate (6.53)	0.055 ± 0.008 ^a^	0.073 ± 0.011 ^a^	0.065 ± 0.013 ^a^
Lactate (1.33)	2.310 ± 0.178 ^a^	3.800 ± 0.133 ^ab^	4.658 ± 0.788 ^b^
***Other compounds***			
Ac-carnitine (3.20)	0.063 ± 0.002 ^a^	0.217 ± 0.009 ^b^	0.166 ± 0.012 ^c^
AMP (8.28)	0.246 ± 0.009 ^a^	0.696 ± 0.049 ^b^	0.613 ± 0.059 ^b^
Carnitine (3.24)	0.135 ± 0.009 ^a^	0.198 ± 0.009 ^b^	0.212 ± 0.016 ^b^
Creatine (3.94)	3.053 ± 0.102 ^a^	8.026 ± 0.411 ^b^	7.869 ± 0.596 ^b^
Glucose (3.26 and 5.25)*	28.108 ± 2.356 ^a^	30.625 ± 2.996 ^a^	36.422 ± 3.901 ^a^
*Myo*-inositol (3.65)	10.325 ± 0.768 ^a^	11.362 ± 0.804 ^a^	13.650 ± 1.787 ^a^
**Lipid extract**			
	**mol % (n = 3)**	**mol % (n = 3)**	**mol % (n = 3)**
CHO (0.74)	8.223 ± 0.272 ^a^	7.451 ± 0.750 ^a^	8.292 ± 0.698 ^a^
SFA	32.602 ± 1.283 ^ab^	29.920 ± 2.870 ^a^	39.489 ± 1.964 ^b^
DUFA (2.81)	3.605 ± 0.115 ^a^	3.710 ± 0.206 ^a^	4.873 ± 0.115 ^b^
UFA (2.08)	53.180 ± 1.153 ^a^	56.354 ± 1.948 ^a^	47.585 ± 1.242 ^b^
PUFA (2.86)	31.557 ± 0.416 ^a^	31.507 ± 1.073 ^a^	20.969 ± 1.228 ^b^
PC (3.28)	21.194 ± 0.736 ^a^	15.236 ± 1.937 ^b^	17.987 ± 0.093 ^ab^
PE (3.21)	12.101 ± 0.137 ^a^	12.321 ± 1.040 ^a^	10.786 ± 0.370 ^a^
SMN (5.76)	5.994 ± 0.187 ^a^	6.274 ± 0.223 ^a^	4.635 ± 0.832 ^a^

^a, b, c^ Different superscript letters within the same row indicate a significant difference (P < 0.05).

^ab^ This label indicates no significant differences with a and b within the same row.

* β- and α- anomers, respectively.

*Abbreviations—*Ac-carnitine: acetylcarnitine; AMP: adenosine monophosphate; CHO: cholesterol; SFA: total content of saturated fatty acids; DUFA: diunsaturated fatty acids; UFA: total content of unsaturated fatty acids; PUFA: polyunsaturated fatty acid; PC: phosphatidylcholine; PE: phosphatidylethanolamine; SMN: sphingomyelin.

As regard water-soluble metabolites, different classes of compounds including amino acids, organic acids, and others were assigned. The amount of the majority of sperm amino acids was significantly affected by the age of male turkeys; whereas only Ala, Gly, Glu and Gln did not show significant differences between ages. The amount of Ile, Phe, Leu, Tyr and Val at 32 weeks of age was significantly higher compared to that of the same compounds at later ages. In contrast, the amount of Asp in turkey sperm showed an opposite trend during ageing and the highest significant value was found at 56 weeks of age (Table 1).

A general increase in the content of all other components identified in the water extract was found in turkey sperm during ageing. The sperm content of creatine, carnitine and adenosine monophosphate (AMP) significantly increased from 32 to 44 weeks and no significant differences were found between 44 and 56 weeks. Acetylcarnitine (ac-carnitine) concentration also increased with respect to 32 weeks with significant differences found between all ages. Among the organic acids, lactate was the most abundant and a progressive increase of its concentration was found in turkey sperm, although only the values at 32 and 56 weeks of age were significantly different. No significant differences were found for other organic acids as well as for glucose and *myo*-inositol sperm content (Table 1).

As concern the lipid extract, the metabolites identified in ^1^H spectra are reported in Table 1. Using ^1^H NMR spectra it is possible to assign the signals of different classes of fatty acids, namely saturated (SFA), diunsaturated (DUFA), polyunsaturated (PUFA) and all unsaturated fatty acids (UFA), but it is impossible to distinguish individual fatty acids of the same class. DUFA and SFA level significantly increased at 56 weeks of age, whereas both UFA and PUFA content significantly decreased at 56 weeks of age. As to cholesterol (CHO), its content in turkey sperm showed a low variability and no significant changes were found at different ages of the males.

Among different phospholipid classes, the sperm content of PC significantly decreased from 32 to 44 weeks of age and no further changes were found at 56 weeks. In contrast, the sperm content of PE and SMN was not significantly affected by bird age.

### Correlation

Pearson correlation coefficients were calculated in order to study the correlation between NMR measured metabolites and sperm quality parameters (Table 2).

**Table 2:** Pearson correlation between sperm qualitative parameters and metabolites identified in turkey fresh semen, at 32, 44 and 56 weeks of male age.

Metabolite	Sperm variables			
	Concentration	Mobility	Viability	Osmotic tolerance
Asp		-0.678**		-0.724**
Glu				-0.663*
Gly				-0.674*
Ile	0.753**			
Leu	0.807**			
Phe	0.844*			
Tyr	0.786**			
Val	0.809**			
Citrate				-0.603*
Formate				-0.635*
AMP				-0.598*
Carnitine		-0.611*		
Creatine		-0.601*		-0.602*
Glucose		-0.540*		-0.690**
Lactate		-0.689**		-0.802**
*Myo*-inositol		-0.553*		-0.736**
DUFA	-0.907**			
PUFA	0.903**			
Smn			0.676*	

Pearson correlation coefficients were calculated for the sperm qualitative parameters (Fig 1) *versus* metabolites detected by NMR (Table 1).

Only significant correlation values

* at the 0.05 level

** at the 0.01 level are reported.

*Abbreviations—*Ac-carnitine: acetylcarnitine; AMP: adenosine monophosphate; DUFA: diunsaturated fatty acids.

Sperm concentration was positively correlated with Ile, Leu, Val, Tyr, PUFA (P<0.01) and Phe (P < 0.05) and negatively correlated with DUFA (P < 0.01).

Mobility was negatively correlated with several metabolites identified in the water extract: Asp, lactate (P < 0.01), carnitine, creatine, myo-inositol and glucose (P < 0.05). The proportion of viable sperm was negatively correlated with Smn (P < 0.05) and no correlations were found with the water-soluble metabolites. SOT was negatively correlated with the major part of water-soluble metabolites, whereas no significant correlations were found between SOT and the different lipids. In particular, significant negative correlation were calculated between SOT and Asp, Glu, lactate, glucose and myo-inositol (P < 0.01), Gly, creatine, citrate, formate and AMP (P < 0.05) (Table 2).

## Discussion

This study was designed to evaluate the changes in the metabolite profile of turkey spermatozoa during the reproductive period and their influence on semen quality. Thus, the metabolic profile, assessed for the first time by NMR analysis, and semen quality parameters of turkey male breeders at 32, 44 and 56 weeks of age were evaluated.

The measured semen quality parameters reported here are in agreement with a general decrease in sperm quality occurred with ageing already observed in turkey [9], in other birds and in mammals [30–34]. In fact, the highest values in sperm quality parameters (concentration, mobility and osmotic tolerance) were observed at the beginning of the reproductive period, whereas strongly declined at the end of it. In particular, a reduction in sperm concentration over 2.5 billion of sperm/mL was observed from 32 to 56 weeks of age, and a strong decrease was also detected in both sperm mobility and osmotic tolerance.

The semen quality parameters can be correlated to the variation in the level of several cellular metabolites.

### Water-soluble metabolites

The sperm content in amino acids was higher at the beginning of the reproductive period (32 weeks of age), with the only exception of Asp which increased during ageing. Moreover, positive correlation between number of sperm/mL and the amount of the Ile, Phe, Leu, Val and Tyr was revealed. We can assume that the higher sperm quality found in 32 weeks old turkeys is somehow related to the higher concentration of these amino acids in sperm.

In contrast, the highest content of Asp in sperm was scored in samples collected from 56 weeks old turkeys, in concomitance with the lowest sperm quality. This leads us to hypothesize that the higher levels of Asp, due to male ageing, are somehow related to a decrease in sperm quality, in particular in sperm mobility and osmotic tolerance, as substantiated by the negative correlation found among Asp and these sperm qualitative parameters. Note that NMR analysis gives the sum of L and D- enantiomers of Asp. The increase of L-Asp could be explained by the increase in oxaloacetate level which, via glutammic-oxaloacetic transaminase [35], can be converted into L-Asp in an almost equilibrium reaction. On the other hand, D-Asp levels were shown to increase in mice testis during development [36].

Although authors reported that Ala concentration affected stallion sperm motility and viability [37], here no change in Ala sperm content was found despite the sperm quality decrease during the reproductive period.

Among the other water-soluble metabolites, a significant increase of both carnitine and ac-carnitine content in the sperm was found from 32 to 56 weeks of age, and the carnitine level was also negatively correlated with mobility. L-carnitine plays a powerful role in the process of sperm formation, sperm maturation, and the maintenance of sperm quality [38, 39]. Moreover, L-carnitine can also be involved in energy metabolic processes, i.e. β-oxidation, following ejaculation. Proteomic investigations confirmed the occurrence of β-oxidation enzymes in both human [40] and stallion [41] sperm. Consistently, the addition of L-carnitine to extenders improves the quality of stored sperm in mammalian [42, 43] and avian species [44]. In contrast with the literature, our results show a negative association between carnitine and a sperm fundamental function as mobility. Taking in consideration the high oxidative metabolism of turkey sperm [45–47], we suggest that the increase in both carnitine and ac-carnitine content measured at 44 and 56 weeks of age could be associated to a reduction in mitochondrial metabolism occurring during ageing. Namely, if mitochondrial β-oxidation and/or further catabolism of its main product (Acetyl-CoA) is progressively impaired, less substrates will be used and as a consequence their cellular content will progressively increase over time. In this regard, mitochondria have been reported to be highly susceptible to ageing and proposed to be “a common link between ageing and fertility loss” [48]. In turkeys, the biostimulation of mitochondria, as a result of He-Ne laser irradiation of semen, increases the energetic charge of sperm with a consequent slower decrease in sperm quality during storage [45].

The increase in the lactate content, doubled at 56 weeks of age, can be also associated with a reduced mitochondrial metabolism. It is noteworthy that lactate, far from being only a side product of glycolysis, plays an active role in sperm bioenergetics [49]. In particular, the occurrence of a mitochondrial L-lactate dehydrogenase [50] allows mitochondria to actively metabolize this substrate with NADH production inside cellular matrix [49]. Thus, a loss in mitochondrial efficiency in utilizing L-lactate as energy substrate could lead to lactate intracellular increase. As a consequence, less energy would be available to perform sperm motion, as substantiated by the negative correlation between lactate and the sperm mobility (see Table 2). In addition, the increase of L-lactate intracellular level could resemble the scenario recently reported by Matsuzaki et al. [51]: the L-lactate, produced by oviductal sperm storage tubules, enters spermatozoa with a consequent cytoplasmic acidification resulting in dynein ATPase inactivation and the flagellar quiescence.

Increased creatine amount was found at 44 and 56 weeks of age. Since this metabolite is negatively correlated with SOT and mobility, we can assume that creatine is related to a loss in sperm integrity and function occurring during the reproductive period. This result appears rather unexpected in the light of the role played by creatine in the transfer and storage of cell energy, especially in sperm [52]. However, the increased creatine content could also derive from an increased availability of Arg and Gly. In fact, in Sertoli cells, arginine can transfer the guanidine group to Gly to form guanidinoacetic acid, which is the precursor of creatine [53]. In this regard we measured very high levels of Gly in turkey sperm, but unfortunately we could not measure arginine levels (due to overlapping of Arg ^1^H signals with more intense signals from other metabolites) to further substantiate this hypothesis.

As known, AMP is a signal molecule of a reduced energetic state of the cell, and its content was found to increase at 44 and 56 weeks of age, thus somehow supporting the hypothesis of a reduced energy availability in turkey sperm ageing. Thus, in the light of these high levels of AMP we suggest that the reduction in sperm quality could be also related to altered activity of AMP-activated kinase (AMPK) a sensor of energy metabolism in the cells. It is known that AMPK plays an active role in sperm functions [54], especially in avian species where a decreased sperm motility has been found as a result of AMPK inhibition [55]. Thus, we could hypothesize that AMPKs expression and/or activity is somehow reduced as the reproductive age progressed; however, this aspect needs to be elucidated in future studies.

Thus, summarizing what reported in this section, our results depict a clear scenario in which mitochondrial metabolism is reduced in turkey sperm during ageing.

### Lipids

Before discussing our results on lipid extract, it is appropriate to give a general overview about peculiarity of the turkey sperm lipids. As already known, lipids are basic component of spermatozoa since they contribute to the structure of plasma membrane and are involved in vital aspects of cell metabolism and function [10]. Moreover, sperm lipids have been considered as a preferential substrate for energy production during *in vitro* semen storage under aerobic conditions in birds [56, 57].

Avian sperm membrane lipids are composed primarily of free cholesterol (10–30%) and phospholipids (60–80%) whose composition is unique due to the unusual high content of long chain polyunsatured fatty acids (LC-PUFA) [58–60]. Moreover, sperm PUFA composition is specie-specific, and turkey spermatozoa are characterized by high proportions of *n*-6 and *n*-9 PUFA [60]. According to our results the levels of PUFA measured in sperm of 32 weeks old turkeys are similar to those previously reported in the same species [8] and in goose also [61], whereas lower levels have been reported in other avian species [61].

A significant decrease of sperm PUFA and UFA, in turkey breeders from 32 to 56 weeks of age is in agreement with previous studies showing a general decrease in PUFA during ageing in both chicken [32, 62] and turkey sperm [34]. The loss of sperm quality observed in turkeys during ageing (32–56 weeks) could be also related to a membrane destabilization originating from PUFAs peroxidation as reported in [34].

High amounts of LC-PUFAs in sperm membrane phospholipids are known to contribute to membrane fluidity and flexibility [63], regulation of cellular movement, lipid metabolism, and fusion capacity [64].

Moreover, a higher content in DUFA and a concomitant lower content in PUFA were found at the end of the reproductive period. The major LC-PUFAs found in turkey sperm are arachidonic (20:4n-6) and docosatetraenoic (22:4n-6) acids [8] derived from the same essential precursor linoleic acid (18:2n-6). The changes observed in sperm fatty acid composition during ageing may be related to a reduction of the enzymatic activities involved in the biosynthesis of the peculiar LC-PUFAs from linoleic acid that, in turn, results more available. In the chicken, the dietary supplementation of the n-3 essential precursor linolenic acid has been efficient in increasing the n-3 LC-PUFA sperm content in a clear age dependent way [27].

In conclusion, our results strongly suggest that the decreased semen quality in turkey male ageing is related to a reduction in mitochondrial activity; therefore, any strategy aimed to sustain the high efficiency of mitochondrial metabolism as longer as possible during the whole reproductive period would be desirable.

## Supporting information

S1 Table Summary of water-soluble metabolites identified in the 600.13 MHz ^1^H spectra of fresh spermatozoa from turkey male. (DOC)

S2 Table Summary of liposoluble metabolites identified in the 600 MHz ^1^H spectrum of fresh spermatozoa from turkey male. (DOC)

S3 Table Water soluble metabolite content (mM) in fresh spermatozoa from turkey male. *Abbreviations—*Ac-carnitine: acetylcarnitine; AMP: adenosine monophosphate.
(DOC)

S4 Table Molar % of lipids in fresh spermatozoa from turkey male. *Abbreviations—*CHO: cholesterol; SFA: total content of saturated fatty acids; DUFA: diunsaturated fatty acids; UFA: total content of unsaturated fatty acids; PUFA: polyunsaturated fatty acid; PC: phosphatidylcholine; PE: phosphatidylethanolamine; SMN: sphingomyelin.
(DOC)
