# Supplementary material for: Age-dependent changes in metabolic profile of turkey spermatozoa as assessed by NMR analysis
Source: PLoS One. 2018 Mar 13;13(3):e0194219. doi: 10.1371/journal.pone.0194219 (PMC5849324; doi:10.1371/journal.pone.0194219)
Supplement: S1 Table — (DOC) [file pone.0194219.s001.doc]

**S1 Table**

Summary of water-soluble metabolites identified in the 600.13 MHz ^1^H spectra of fresh spermatozoa from turkey male.

| **Compound** | **Assignment** | **^1^H (ppm)** | **Multeplicity: *J*(Hz)** | **^13^C (ppm)** |
| --- | --- | --- | --- | --- |
| *Carbohydrates* |  |  |  |  |
|  |  |  |  |  |
| α-Glucose | CH-1 | 5.25 | d [3.8] | 93.1 |
|  | CH-2 | 3.55 | dd [9.8;3.8] | 72.5 |
|  | CH-3 | 3.72 |  | 73.7 |
|  | CH-4 | 3.42 |  | 70.6 |
|  | CH-5 | 3.84 |  | 72.4 |
|  | CH_2_-6,6' | 3.84;3.78 |  | 61.6 |
|  |  |  |  |  |
| β-Glucose | CH-1 | 4.66 | d [7.9] | 96.9 |
|  | CH-2 | 3.26 | dd [9.3;8.0] | 75.1 |
|  | CH-3 | 3.51 | t [9.1] | 76.7 |
|  | CH-4 | 3.41 |  | 70.6 |
|  | CH-5 | 3.48 |  | 76.9 |
|  | CH_2_-6,6' | 3.90;3.74 |  | 61.7 |
|  |  |  |  |  |
| Myo-inositol | CH-1 | 4.08 | t [2.9] | 73.2 |
|  | CH-2,5 | 3.56 |  | 72.1 |
|  | CH-3,6 | 3.63 | t [9.7] | 73.4 |
|  | CH-4 | 3.30 | t [9.4] | 75.3 |
|  |  |  |  |  |
| *Organic acids* |  |  |  |  |
|  |  |  |  |  |
| Acetic acid | α-CH_3_ | 1.93 | s | 24.2 |
|  |  |  |  |  |
| Citric acid | α,γ-CH | 2.56 | d [15.6] | 46.1 |
|  | α',γ'-CH | 2.70 | d [15.6] | 46.1 |
|  |  |  |  |  |
| Formic acid | HCOOC | 8.46 |  |  |
|  |  |  |  |  |
| Fumaric acid | CH | 6.52 | s |  |
|  |  |  |  |  |
| Lactic acid | β-CH_3_ | 1.34 | d [6.9] | 21.1 |
|  | α-CH | 4.12 | q [6.9] | 69.5 |
|  |  |  |  |  |
| *Amino acids* |  |  |  |  |
|  |  |  |  |  |
| Alanine | α-CH | 3.80 |  | 51.5 |
|  | β-CH_3_ | 1.49 | d [7.3] | 17.2 |
|  |  |  |  |  |
| Aspartate | α-CH | 3.91 |  | 53.2 |
|  | β-CH | 2.72 |  | 37.5 |
|  | β'-CH | 2.81 | dd [3.9;17.5] | 37.5 |
|  |  |  |  |  |
| Glycine | α-CH_2_ | 3.57 | s | 42.4 |
|  |  |  |  |  |
| Glutamate | α-CH | 3.77 |  | 55.5 |
|  | β-CH | 2.13 |  | 27.9 |
|  | β’-CH | 2.07 |  | 27.9 |
|  | γ-CH_2_ | 2.36 | m | 34.4 |
|  |  |  |  |  |
| Glutamine | α-CH | 3.79 |  | 55.1 |
|  | β-CH_2_ | 2.15 | m | 27.3 |
|  | γ-CH | 2.46 | m | 31.8 |
|  |  |  |  |  |
| Isoleucine | α-CH | 3.68 |  |  |
|  | β-CH | 1.98 |  |  |
|  | γ-CH | 1.26 |  |  |
|  | γ'-CH | 1.47 |  |  |
|  | γ-CH_3_ | 1.01 | d [7.0] |  |
|  | δ-CH_3_ | 0.94 | t [7.3] |  |
|  |  |  |  |  |
| Leucine | α-CH | 3.74 |  |  |
|  | β-CH_2_ | 1.71 |  | 40.7 |
|  | δ-CH_3_ | 0.97 | d [6.1] | 23.0 |
|  | δ'-CH_3_ | 0.96 | d [6.0] | 22.0 |
|  |  |  |  |  |
| Phenylalanine | CH-2,6, ring | 7.34 |  |  |
|  | CH-3,5, ring | 7.43 |  |  |
|  | CH-4, ring | 7.39 |  |  |
|  |  |  |  |  |
| Tyrosine | CH-2,6 | 7.20 | d(8) | 131.8 |
|  | CH-3,5 | 6.91 | d(8) | 116.8 |
|  |  |  |  |  |
| Valine | α-CH | 3.62 |  | 61.4 |
|  | β-CH | 2.27 |  |  |
|  | γ-CH_3_ | 1.00 | d [7.1] |  |
|  | γ'-CH_3_ | 1.05 | d [7.1] | 18.9 |
|  |  |  |  |  |
| *Other metabolites* |  |  |  |  |
|  |  |  |  |  |
| Creatine | N-CH_3_ | 3.04 | s | 38.0 |
|  | N-CH_2_ | 3.94 | s | 54.9 |
|  | N=C |  |  | 158.0 |
|  | COOH |  |  | 175.7 |
|  |  |  |  |  |
| Carnitine | α-CH_2_ | 2.45 |  | 44.0 |
|  | β-CH | 4.57 |  | 65.1 |
|  | γ-CH_2_ | 3.44 |  |  |
|  | N-CH_3_ | 3.23 | s | 55.1 |
|  |  |  |  |  |
| O-acetylcarnitine | α-CH_2_ | 2.53; 2.64 |  |  |
|  | β-CH | 5.61 |  |  |
|  | γ-CH_2_ | 3.61; 3.86 |  |  |
|  | N-CH_3_ | 3.20 | s | 54.9 |
|  |  |  |  |  |
| AMP | CH-2, aromatic ring | 8.55 | s |  |
|  | CH -8, aromatic ring | 8.28 | s |  |
|  | CH-1’, ribose | 6.15 | d [5.6] |  |
|  | CH-2’, ribose | 4.78 |  |  |
|  | CH-3’, ribose | 4.51 |  |  |
|  | CH-4’, ribose | 4.38 |  |  |
|  | CH-5’, ribose | 4.07 |  |  |
