# Supplementary material for: Age-dependent changes in metabolic profile of turkey spermatozoa as assessed by NMR analysis
Source: PLoS One. 2018 Mar 13;13(3):e0194219. doi: 10.1371/journal.pone.0194219 (PMC5849324; doi:10.1371/journal.pone.0194219)
Supplement: S2 Table — (DOC) [file pone.0194219.s002.doc]

**S2 Table.** Summary of liposoluble metabolites identified in the 600 MHz ^1^H spectrum of fresh spermatozoa from turkey male

| **Assignment** | **^1^H, (ppm)** | **Multiplicity: *J* (Hz)** | **^13^C, (ppm)** |
| --- | --- | --- | --- |
| **All fatty acid chains** | | | |
| CH_3_ | 0.92 | t [7.3] | 14.1 |
| CH_2_ | 1.34-1.31 |  |  |
| CH_2_-β | 1.65 |  | 25.3 |
| CH_2_-α | 2.37 |  | 34.4 |
| CH_2_-CH=CH | 2.11 - 2.07 |  | 27.6 |
| CH=CH | 5.41-5.37 |  | 130.3 -128.4 |
| CH_2_-2 | 2.36 | t (7.6) | 35.0 |
| **Linolenic acid chains** | | | |
| CH_2_-11,14 | 2.85 | t (6.0) | 25.9 |
| **Linoleic acid chains** | | | |
| CH_2_-11 | 2.81 | t (6.9) | 26.0 |
| **Phosphatidylcholine** | | | |
| (CH_3_)_3_N | 3.27 | s | 54.3 |
| CH_2_OP | 4.40 |  | 60.4 |
| CH_2_N | 3.72 |  | 66.6 |
| CH_2_ sn3 | 4.13 |  | 64.9 |
| CH_2_ sn1 | 4.46; 4.22 |  | 62.8 |
| CH sn2 | 5.30 |  | 70.7 |
| **Phosphatidylethanolamine** | | | |
| CH_2_OP | 4.18 |  | 63.0 |
| CH_2_N | 3.23 |  | 40.6 |
| CH sn2 | 5.295 |  | 70.0 |
| **Cholesterol** | | | |
| CH_2_-1 | 1.90; 1.12 |  | 37.8 |
| CH_2_-2 | 1.84; 1.53 |  | 31.5 |
| CH-3 | 3.47 |  | 71.7 |
| CH_2_-4 | 2.27 |  | 42.2 |
| CH-6 | 5.37 |  | 121.9 |
| CH_2_-7 | 2.01; 1.54 |  | 32.3 |
| CH-8 | 1.50 |  | 32.4 |
| CH-9 | 0.97 |  | 50.9 |
| CH_2_-11 | 1.55 |  | 21.6 |
| CH_2_-12 | 2.06; 1.21 |  | 40.4 |
| CH-14 | 1.05 |  | 57.2 |
| CH_2_-15 | 1.62; 1.11 |  | 24.7 |
| CH_2_-16 | 1.87; 1.31 |  | 28.7 |
| CH-17 | 1.14 |  | 56.8 |
| CH_3_-18 | 0.74 |  | 12.0 |
| CH_3_-19 | 1.06 |  | 19.6 |
| CH-20 | 1.41 |  | 36.3 |
| CH_3_-21 | 0.97 |  | 18.9 |
| CH_2_-22 | 1.39; 1.04 |  | 36.7 |
| CH_2_-23 | 1.39; 1.20 |  | 24.3 |
| CH_2_-24 | 1.17 |  | 40.0 |
| CH-25 | 1.56 |  | 28.5 |
| CH_3_-26 | 0.91 |  | 22.8 |
| **Sphingomyelin** | | | |
| CH-1 | 5.76 |  | 135.1 |
| CH_2_-10 | 2.22 |  | 36.8 |
| CH_2_-11 | 2.07 |  | 32.9 |
| CH-2 | 5.49 |  | 129.9 |
| CH_2_-4 | 4.19; 4.12 |  | 66.3 |
| CH-5 | 4.07 |  | 71.8 |
| CH-7 | 4.03 |  | 54.3 |
| N(CH_3_)_3_ | 3.27 |  | 54.2 |
