# Supplementary material for: Age-dependent changes in metabolic profile of turkey spermatozoa as assessed by NMR analysis
Source: PLoS One. 2018 Mar 13;13(3):e0194219. doi: 10.1371/journal.pone.0194219 (PMC5849324; doi:10.1371/journal.pone.0194219)
Supplement: S3 Table — Abbreviations—Ac-carnitine: acetylcarnitine; AMP: adenosine monophosphate. (DOC) [file pone.0194219.s003.doc]

**S3 Table**. Water soluble metabolite content (mM) in fresh spermatozoa from turkey male

|  | Age, weeks | 32 | | | | | 44 | | | | 56 | | | | |
| --- | --- | --- | --- | --- | --- | --- | --- | --- | --- | --- | --- | --- | --- | --- | --- |
|  | Sample | 1 | 2 | 3 | 4 | 5 | 1 | 2 | 3 | 4 | 1 | 2 | 3 | 4 | 5 |
| Ala |  | 0.268 | 0.280 | 0.314 | 0.309 | 0.327 | 0.357 | 0.354 | 0.333 | 0.340 | 0.374 | 0.271 | 0.342 | 0.265 | 0.238 |
| Asp |  | 0.426 | 0.382 | 0.404 | 0.388 | 0.391 | 0.485 | 0.521 | 0.511 | 0.587 | 1.024 | 0.656 | 0.726 | 0.591 | 0.443 |
| Gln |  | 1.788 | 1.451 | 2.612 | 2.870 | 5.569 | 3.250 | 3.282 | 2.192 | 2.245 | 3.860 | 2.531 | 2.836 | 2.011 | 1.548 |
| Glu |  | 90.901 | 99.937 | 120.152 | 123.787 | 131.539 | 104.216 | 142.752 | 104.879 | 94.214 | 173.654 | 131.808 | 159.100 | 120.689 | 81.688 |
| Gly |  | 6.221 | 6.676 | 8.738 | 8.732 | 9.316 | 7.743 | 10.656 | 7.784 | 6.402 | 12.955 | 9.621 | 11.906 | 8.646 | 5.764 |
| Ile |  | 0.021 | 0.022 | 0.029 | 0.029 | 0.022 | 0.013 | 0.016 | 0.016 | 0.018 | 0.025 | 0.016 | 0.022 | 0.013 | 0.011 |
| Leu |  | 0.080 | 0.088 | 0.104 | 0.099 | 0.085 | 0.051 | 0.054 | 0.051 | 0.065 | 0.081 | 0.056 | 0.070 | 0.045 | 0.031 |
| Phe |  | 0.034 | 0.035 | 0.039 | 0.041 | 0.037 | 0.029 | 0.030 | 0.025 | 0.027 | 0.035 | 0.025 | 0.029 | 0.024 | 0.015 |
| Tyr |  | 0.067 | 0.072 | 0.093 | 0.090 | 0.079 | 0.024 | 0.041 | 0.033 | 0.062 | 0.062 | 0.046 | 0.059 | 0.033 | 0.021 |
| Val |  | 0.054 | 0.058 | 0.073 | 0.072 | 0.059 | 0.045 | 0.048 | 0.046 | 0.052 | 0.062 | 0.049 | 0.056 | 0.044 | 0.033 |
| Acetate |  | 0.357 | 0.642 | 1.015 | 1.022 | 1.254 | 0.436 | 1.256 | 0.446 | 0.246 | 1.108 | 0.371 | 1.119 | 0.334 | 0.036 |
| Citrate |  | 0.108 | 0.128 | 0.165 | 0.209 | 0.150 | 0.213 | 0.257 | 0.234 | 0.196 | 0.369 | 0.192 | 0.303 | 0.158 | 0.084 |
| Formate |  | 0.049 | 0.047 | 0.065 | 0.087 | 0.093 | 0.155 | 0.250 | 0.074 | 0.050 | 0.182 | 0.128 | 0.193 | 0.076 | 0.017 |
| Fumarate |  | 0.039 | 0.033 | 0.060 | 0.073 | 0.071 | 0.086 | 0.098 | 0.048 | 0.060 | 0.100 | 0.059 | 0.088 | 0.049 | 0.029 |
| Lactate |  | 2.135 | 1.822 | 2.433 | 2.260 | 2.902 | 3.958 | 3.912 | 3.401 | 3.926 | 6.353 | 3.663 | 6.530 | 4.305 | 2.438 |
| Ac-Carnitine | | 0.057 | 0.059 | 0.068 | 0.067 | 0.063 | 0.234 | 0.193 | 0.212 | 0.231 | 0.206 | 0.133 | 0.162 | 0.167 | 0.164 |
| AMP |  | 0.226 | 0.227 | 0.275 | 0.253 | 0.250 | 0.808 | 0.697 | 0.570 | 0.711 | 0.737 | 0.592 | 0.760 | 0.517 | 0.459 |
| Carnitine |  | 0.103 | 0.147 | 0.145 | 0.151 | 0.129 | 0.200 | 0.175 | 0.201 | 0.216 | 0.252 | 0.216 | 0.241 | 0.167 | 0.184 |
| Creatine |  | 2.825 | 3.187 | 3.275 | 3.191 | 2.787 | 8.299 | 6.805 | 8.433 | 8.569 | 9.284 | 7.455 | 9.277 | 6.834 | 6.499 |
| Glucose |  | 20.984 | 24.031 | 32.442 | 32.330 | 30.760 | 27.971 | 39.246 | 29.704 | 25.582 | 45.784 | 36.693 | 42.840 | 33.299 | 23.499 |
| Myo-Inositol | | 8.243 | 8.741 | 11.127 | 11.415 | 12.099 | 11.114 | 13.626 | 10.867 | 9.843 | 18.565 | 12.911 | 16.284 | 12.367 | 8.123 |
