# Supplementary material for: Age-dependent changes in metabolic profile of turkey spermatozoa as assessed by NMR analysis
Source: PLoS One. 2018 Mar 13;13(3):e0194219. doi: 10.1371/journal.pone.0194219 (PMC5849324; doi:10.1371/journal.pone.0194219)
Supplement: S4 Table — Abbreviations—CHO: cholesterol; SFA: total content of saturated fatty acids; DUFA: diunsaturated fatty acids; UFA: total content of unsaturated fatty acids; PUFA: polyunsaturated fatty acid; PC: phosphatidylcholine; PE: phosphatidylethanolamine; SMN: sphingomyelin. (DOC) [file pone.0194219.s004.doc]

**S4 Table**. Molar % of lipids in fresh spermatozoa from turkey male

| Age, week | Sample | CHO | UFA | DUFA | PC | PE | Smn | SFA | PUFA |
| --- | --- | --- | --- | --- | --- | --- | --- | --- | --- |
| 32 | 1 | 8.529 | 50.961 | 3.379 | 21.129 | 12.356 | 5.705 | 34.805 | 30.803 |
| 32 | 2 | 7.682 | 53.747 | 3.682 | 22.500 | 12.057 | 5.933 | 32.638 | 31.631 |
| 32 | 3 | 8.460 | 54.833 | 3.753 | 19.954 | 11.888 | 6.345 | 30.362 | 32.239 |
| 44 | 1 | 8.946 | 60.108 | 3.625 | 13.343 | 14.355 | 6.681 | 24.265 | 33.419 |
| 44 | 2 | 6.806 | 55.383 | 4.102 | 19.107 | 11.681 | 5.913 | 31.898 | 29.706 |
| 44 | 3 | 6.601 | 53.571 | 3.402 | 13.257 | 10.928 | 6.229 | 33.599 | 31.395 |
| 56 | 1 | 8.092 | 45.136 | 4.646 | 18.064 | 11.297 | 3.357 | 43.415 | 23.364 |
| 56 | 2 | 7.195 | 49.174 | 4.956 | 18.096 | 10.067 | 6.197 | 37.434 | 20.241 |
| 56 | 3 | 9.588 | 48.444 | 5.018 | 17.801 | 10.993 | 4.351 | 37.617 | 19.301 |
